# The complete chloroplast genome sequence of *Quercus myrsinifolia* (Fagaceae)

**DOI:** 10.1080/23802359.2019.1667276

**Published:** 2019-09-20

**Authors:** Yao Li, Lu Wang, Yanming Fang

**Affiliations:** Co-Innovation Center for Sustainable Forestry in Southern China, College of Biology and the Environment, Key Laboratory of State Forestry and Grassland Administration on Subtropical Forest Biodiversity Conservation, Nanjing Forestry University, Nanjing, China

**Keywords:** chloroplast genome, Fagaceae, phylogeny, *Quercus myrsinifolia*

## Abstract

*Quercus myrsinifolia* Blume is an evergreen oak tree species native to East Asia, and is also one of the dominant trees of subtropical evergreen broad-leaved forests. In this study, we sequenced and analysed the complete chloroplast (cp) genome of the species. The circular genome is 160,803 bp in size, consisting of two copies of inverted repeat (IR) regions of 25,840 bp, one large single-copy (LSC) region of 90,223 bp, and one small single-copy (SSC) region of 18,900 bp. It encodes a total of 114 unique genes, including 80 protein-coding genes, 30 tRNA genes, and four rRNA genes. Phylogenetic analysis based on 30 cp genome sequences indicated that *Q. myrsinifolia* was among the members of section *Cyclobalanopsis*, and was most closely related to *Q. sichourensis*.

*Quercus myrsinifolia* Blume is an evergreen oak tree species native to East Asia. It is widely distributed across China, Japan, Korea, Laos, Thailand, and Vietnam, and grows at an altitude ranging from 200 m to 2500 m above sea level. As one of the dominant trees of subtropical evergreen broad-leaved forests, it is of ecological importance in maintaining mountain ecosystem, and providing habitats and foods for various species, including endangered animals like Tibetan macaque (*Macaca thibetana*) (You et al. 2013). It is also an important commercial tree species for producing excellent timber. Previous phylogenomic studies have shown that *Q. myrsinifolia* belongs to the compound trichome base (CTB) lineage within *Quercus* section *Cyclobalanopsis* (Deng et al. 2018). However, the plastid genome sequence of the species remains unknown. In this study, we report the complete chloroplast (cp) genome of *Q. myrsinifolia* based on Illumina paired-end sequencing data.

Fresh young leaves were sampled from an adult tree growing at the Huangshan Mountain, Anhui Province, China (30.10°N,118.17°E). The voucher specimen (accession number YL20190417008) was preserved at the Herbarium of Nanjing Forestry University (HNFU). Total DNA extraction and whole genome sequencing on the Illumina Hiseq X Ten platform were conducted by Nanjing Genepioneer Biotechnologies Inc. (Nanjing, China). A total of 28,699,387 clean reads were produced and then used for the *de novo* assembly with NOVOplasty 2.7.2 (Dierckxsens et al. 2016). Gene annotation was performed using the CpGAVAS pipeline (Liu et al. 2012).

The complete cp genome of *Q. myrsinifolia* (GenBank accession number MN199025) is a circular molecule of 160,803 bp in length, consisting of two copies of IR (25,840 bp) separated by the LSC (90,223 bp) and SSC (18,900 bp) regions. The overall GC content was 36.89%, while the corresponding values of the LSC, SSC, and IR regions were 34.74%, 31.09%, and 42.77%, respectively. The cp genome encoded a total of 133 genes, of which 114 were unique and 19 were duplicated in the IR regions. The 114 unique genes contained 80 protein-coding genes, 30 tRNA genes, and four rRNA genes. Fifteen genes contained introns, 12 of which (six protein-coding genes and six tRNA genes) contained one intron, and three of which (*rps12*, *ycf3*, and *clpP*) contained two introns.

To identify the phylogenetic position of *Q. myrsinifolia*, we reconstructed a maximum-likelihood (ML) tree using cp genome sequences of 24 Fagaceae species and six outgroups from Juglandaceae and Betulaceae (Figure 1). Three locally collinear blocks (LCBs) were identified and a matrix of 82,255 bp was generated by the HomBlocks pipeline (Bi et al. 2018). Using PartitionFinder 2 (Lanfear et al. 2016), the TVM + G model and the TVM + I + G model were chosen for the subset of LCBs 1-2 (10,255 bp) and the subset of LCB 3 (72,000 bp), respectively. The IQ-tree 1.6.8 program (Nguyen et al. 2014) was used to perform the ML analyses, with branch support values estimated through 20,000 ultrafast bootstrap replicates. Our results indicated that *Q. myrsinifolia* was among the members of section *Cyclobalanopsis*, and was most closely related to *Q. sichourensis* with 99% bootstrap support.

**Figure 1.**
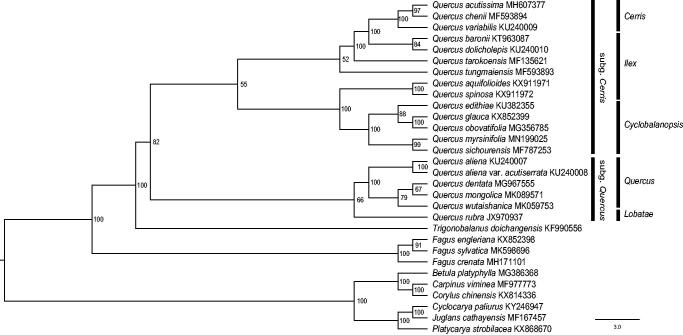
The maximum-likelihood (ML) phylogenetic tree reconstructed by IQ-tree 1.6.8 (Nguyen et al. 2014) based on cp genome sequences of 24 Fagaceae species and six outgroups from Juglandaceae and Betulaceae. The bootstrap support value is labelled for each node. Black bars show two subgenera (*Cerris* and *Quercus*) and five sections (*Cerris*, *Ilex*, *Cyclobalanopsis*, *Quercus*, and *Lobatae*) of *Quercus* (Denk et al. 2017).
